# Large language models for patient education prior to interventional radiology procedures: a comparative study

**DOI:** 10.1186/s42155-025-00609-z

**Published:** 2025-10-13

**Authors:** Bogdan Levita, Semil Eminovic, Willie Magnus Lüdemann, Dirk Schnapauff, Robin Schmidt, Anna-Maria Haack, Andrea Dell’Orco, Jawed Nawabi, Tobias Penzkofer

**Affiliations:** 1 Department of Radiology, Charité – Universitätsmedizin Berlin, Humboldt-Universität Zu Berlin, Freie Universität Berlin, Berlin Institute of Health, Augustenburger Platz 1, 13353 Berlin, Germany; 2 Department of Neuroradiology, Charité – Universitätsmedizin Berlin, Humboldt-Universität Zu Berlin, Freie Universität Berlin, Berlin Institute of Health, Berlin, Germany; 3 Experimental Clinical Research Center (ECRC) at Charité-Universitätsmedizin Berlin and Max-Delbrück-Centrum Für Molekulare Medizin (MDC), Robert-Rössle-Straße 10, 13125 Berlin, Germany; 4 Berlin Institute of Health (BIH), Berlin, Germany

**Keywords:** Large language models, Interventional radiology, Patient education

## Abstract

**Purpose:**

This study evaluates four large language models’ (LLMs) ability to answer common patient questions preceding transarterial periarticular embolization (TAPE), computed tomography (CT)-guided high-dose-rate (HDR) brachytherapy, and bleomycin electrosclerotherapy (BEST). The goal is to evaluate their potential to enhance clinical workflows and patient comprehension, while also assessing associated risks.

**Materials and methods:**

Thirty-five TAPE, 34 CT-HDR brachytherapy, and 36 BEST related questions were presented to ChatGPT-4o, DeepSeek-V3, OpenBioLLM-8b, and BioMistral-7b. The LLM-generated responses were independently assessed by two board-certified radiologists. Accuracy was rated on a 5-point Likert scale. Statistics compared LLM performance across question categories for patient-education suitability.

**Results:**

DeepSeek-V3 attained the highest mean scores for BEST [4.49 (± 0.77)] and CT-HDR [4.24 (± 0.81)] and demonstrated comparable performance to ChatGPT-4o for TAPE-related questions (DeepSeek-V3 [4.20 (± 0.77)] vs. ChatGPT-4o [4.17 (± 0.64)]; *p* = 1.000). In contrast, OpenBioLLM-8b (BEST 3.51 (± 1.15), CT-HDR 3.32 (± 1.13), TAPE 3.34 (± 1.16)) and BioMistral-7b (BEST 2.92 (± 1.35), CT-HDR 3.03 (± 1.06), TAPE 3.33 (± 1.28)) performed significantly worse than DeepSeek-V3 and ChatGPT-4o across all procedures. Preparation/Planning was the only category without statistically significant differences across all three procedures.

**Conclusion:**

DeepSeek-V3 and ChatGPT-4o excelled on TAPE, BEST, and CT-HDR brachytherapy questions, indicating potential to enhance patient education in interventional radiology, where complex but minimally invasive procedures often are explained in brief consultations. However, OpenBioLLM-8b and BioMistral-7b exhibited more frequent inaccuracies, suggesting that LLMs cannot replace comprehensive clinical consultations yet. Patient feedback and clinical workflow implementation should validate these findings.

**Supplementary Information:**

The online version contains supplementary material available at 10.1186/s42155-025-00609-z.

## Introduction

Large language models (LLMs) are progressively being adopted in medicine to aid in clinical decision-support, medical documentation, diagnostics, and patient education, demonstrating both significant promise and emerging risks in healthcare workflows [1–3]. An early benchmark study found that ChatGPT achieved near-passing performance on a radiology board-style examination, indicating the depth of domain-specific knowledge LLMs can provide while highlighting the necessity for validation in specialized settings [4]. They can also offer accurate and detailed responses to patient inquiries in Interventional Radiology (IR), providing new opportunities for patient education by reducing fear and improving compliance, though also posing some risks of misinformation [5–7].

IR has significantly expanded its applications over the last years, largely due to advancements in technology, offering advantages such as reduced risk and shorter recovery times compared to traditional open surgery [8]. However, IR lacks public awareness and understanding by patients: a study found that 65% of patients were unfamiliar with IR and 72% did not recognize IR physicians as such [9]. As a result, IR physicians must overcome a more significant “gap” in educating patients to help them make well-informed decisions.

This gap is particularly evident for less commonly performed procedures, such as Bleomycin electrosclerotherapy (BEST), transarterial periarticular embolization (TAPE), and CT-guided high-dose-rate (HDR) brachytherapy, where patient knowledge is often especially limited.

BEST combines electroporation with bleomycin in the treatment of vascular malformations [10, 11], improving local drug uptake while reducing systemic effects [12], and exhibits promising outcomes in multiple studies [13–15]. TAPE is an increasingly popular therapeutic procedure for chronic joint pain, often when drug and physical therapy options have been exhausted, and can be performed comparatively quickly and inexpensively, contributing to significant pain relief [16–18]. CT-HDR brachytherapy is a minimally invasive procedure for liver tumors, demonstrating favorable outcomes with extended survival and excellent local tumor control in numerous studies [19–21]. However, the three procedures are limited to specialized centers and require comprehensive patient education and informed consent [22], which can be time- and resource-intensive in clinical practice [23, 24]. Despite a growing focus on patient empowerment [25], many patients lack sufficient understanding of medical procedures [24] and their risks [26].

The safety and efficacy of LLMs in educating patients about less common procedures like BEST, TAPE, or CT-HDR brachytherapy remain uncertain, as does their potential to reduce clinicians’ workload, improve patient satisfaction, or support clinical workflows. Given their rapid evolution, this study evaluates the accuracy of four LLMs in answering common patient questions about CT-HDR brachytherapy, TAPE, and BEST, aiming to assess their clinical relevance and ability to enhance patient communication in IR.

## Materials and methods

### Study design

Due to the human-generated sample dataset, ethics committee approval was not required.

In this prospective evaluation study, two radiology residents (fifth and second year, respectively) created 107 questions based on commonly asked patient questions encountered in daily clinical practice before TAPE (35), CT-HDR brachytherapy (34), and BEST (36). These questions were derived from common patient encounters at our institution as well as standard information provided during pre-procedural conversations with patients. “Commonly asked” is characterized as questions repeatedly encountered across multiple patients and consultations. To minimize subjective bias, the residents referenced documented practice patterns and validated the selection together with two board-certified interventional radiologists (18 and 11 years of experience), who ensured clinical relevance and realism.

Responses were generated by four state-of-the-art LLMs, three open-source models (DeepSeek-V3, OpenBioLLM-8b, BioMistral-7b) and one proprietary model (ChatGPT-4o). The two interventional radiologists independently evaluated responses using a 5-point Likert scale (Supplementary Table S1). ChatGPT-4o and DeepSeek-V3 were selected for their public visibility and scientific validation [27, 28]. OpenBioLLM-8b and BioMistral-7b enabled comparison with smaller, medically pre-trained language models.

### Question design and prompting

Question categories were general information, preparation/planning, risks/contraindications, recovery/follow-up, and side effects/complications. Two examples of asked questions:

What medication do I have to stop taking before a TAPE and for how long?

or “What happens if I cannot withstand the radiation during CT-HDR brachytherapy and have to interrupt the treatment?

All four LLMs were prompted identically, beginning with a neutral patient–role introduction:

I am a patient. I am due to have a CT-HDR brachytherapy and have some questions about this procedure. Can you answer each of the following questions in an understandable way, and respectively the same for TAPE and BEST.

To limit wording and content bias, the exact question text was kept fixed across models and leading phrasing was avoided. Each question was submitted once per model, with no retries or manual edits. All questions for each procedure were subsequently submitted via API to all four LLMs (on December 10th 2024) using identical prompts to ensure comparability and consistency. All questions are provided as supplementary material; all responses and ratings are documented and available upon request.

### Response evaluation

Response accuracy from DeepSeek-V3, OpenBioLLM-8b, BioMistral-7b, and ChatGPT-4o was rated by two independent board-certified radiologists using a 5-point Likert scale (1 = Very inaccurate/completely false, very likely to mislead; 2 = Inaccurate/mostly false, likely to mislead; 3 = Neutral/moderately accurate, overall acceptable; 4 = Accurate/mostly correct, only very few inaccuracies, unlikely to mislead; 5 = Very accurate/completely correct, very unlikely to mislead; displayed in Supplementary Table S1) and subsequently statistically compared. The model-generated answers were not revised, edited, or regenerated after initial output. The first complete response returned by the model was recorded and used for evaluation without any modification. No predefined risk tiering of questions or errors was performed. Potential safety concerns identified by reviewers were documented qualitatively. LLMs were anonymized to ensure blinded evaluation by the radiologists.

### Statistical analysis

Categorical and ordinal ratings are presented as counts and percentages (*n*, %), and overall scores are reported as mean ± SD, calculated from the radiologists’ individual means. To assess statistically significant differences between models, a two-tailed, four-sample Friedman test was applied (repeated measures, ordinal scale). *P*-values below 0.05 were considered statistically significant. If so, post hoc Wilcoxon signed-rank tests with Holm correction were used to assess pairwise statistically significant differences between LLM ratings. Holm correction adjusted the significance level for multiple comparisons. Interrater agreement was assessed using the two-way random effects single-measure intraclass correlation coefficient (ICC) and Cohen’s Kappa to evaluate absolute agreement between both raters per model and across all items (Supplementary Table S2). Analyses and visualizations were performed using Python (version 3.9.13) with packages including Pandas (version 2.2.2), NumPy (version 1.23.1), SciPy (version 1.13.1; Scikit-Posthocs version 0.10.0), Matplotlib (version 3.9.2), and Seaborn (version 0.13.2).

## Results

**Fig. 1:**
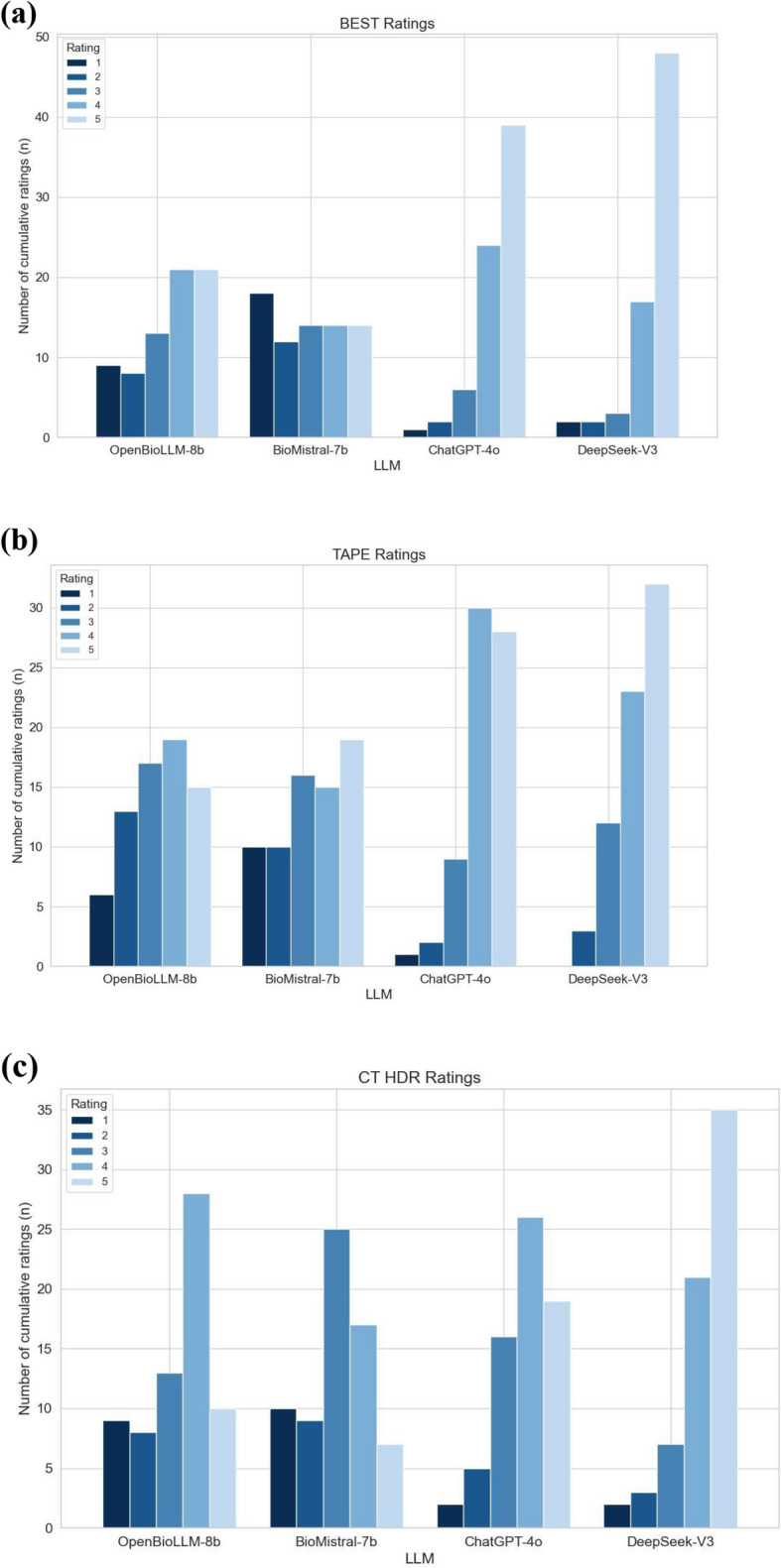
Bar charts that display the cumulative grading of the performance of four LLMs (OpenBioLLM-8b, BioMistral-7b, ChatGPT-4o, DeepSeek-V3) assessed by two radiologists in answering patient questions before the interventional radiology procedures **a** BEST, **b** TAPE, and **c** CT HDR

Performance grading of the four LLMs is displayed in Table 1 (BEST), Table 2 (CT-HDR brachytherapy), and Table 3 (TAPE). Statistics for all questions and all covered categories are illustrated in Table 4. Cumulative ratings from both radiologists are displayed in Fig. 1a–c.

**Table 1:** Grading of the performance of four Large Language Models (OpenBioLLM-8b, BioMistral-7b, ChatGPT-4o, DeepSeek-V3) in answering patient questions before a *BEST* procedure (evaluations of both radiologists combined). With two radiologists grading 36 BEST-related questions, there are 72 ratings in total

Rating	OpenBioLLM-8b	BioMistral-7b	ChatGPT-4o	DeepSeek-V3
5	21 (29.17%)	14 (19.44%)	39 (54.17%)	48 (66.67%)
4	21 (29.17%)	14 (19.44%)	24 (33.33%)	17 (23.61%)
3	13 (18.06%)	14 (19.44%)	6 (8.33%)	3 (4.17%)
2	8 (11.11%)	12 (16.67%)	2 (2.78%)	2 (2.78%)
1	9 (12.50%)	18 (25.00%)	1 (1.39%)	2 (2.78%)
Mean (± SD)	*3.51 (*± *1.15)*	*2.92 (*± *1.35)*	*4.36 (*± *0.71)*	*4.49 (*± *0.77)*

**Table 2:** Grading of the performance of four Large Language Models (OpenBioLLM-8b, BioMistral-7b, ChatGPT-4o, DeepSeek-V3) in answering patient questions before a *CT-HDR* procedure (evaluations of both radiologists combined). With two radiologists grading 34 CT-HDR-related questions, there are 68 ratings in total

Rating	OpenBioLLM-8b	BioMistral-7b	ChatGPT-4o	DeepSeek-V3
5	10 (14.71%)	7 (10.29%)	19 (27.94%)	35 (51.47%)
4	28 (41.18%)	17 (25.00%)	26 (38.24%)	21 (30.88%)
3	13 (19.12%)	25 (36.76%)	16 (23.53%)	7 (10.29%)
2	8 (11.76%)	9 (13.24%)	5 (7.35%)	3 (4.41%)
1	9 (13.24%)	10 (14.71%)	2 (2.94%)	2 (2.94%)
Mean (± SD)	*3.32 (*± *1.25)*	*3.03 (*± *1.18)*	*3.81 (*± *1.03)*	*4.24 (*± *1.01)*

**Table 3:** Grading of the performance of four Large Language Models (OpenBioLLM-8b, BioMistral-7b, ChatGPT-4o, DeepSeek-V3) in answering patient questions before a *TAPE* procedure (evaluations of both radiologists combined). With two radiologists grading 35 TAPE-related questions, there are 70 ratings in total

Rating	OpenBioLLM-8b	BioMistral-7b	ChatGPT-4o	DeepSeek-V3
5	15 (21.43%)	19 (27.14%)	28 (40.00%)	32 (45.71%)
4	19 (27.14%)	15 (21.43%)	30 (42.86%)	23 (32.86%)
3	17 (24.29%)	16 (22.86%)	9 (12.86%)	12 (17.14%)
2	13 (18.57%)	10 (14.29%)	2 (2.86%)	3 (4.29%)
1	6 (8.57%)	10 (14.29%)	1 (1.43%)	0 (0.00%)
Mean (± SD)	*3.34 (*± *1.16)*	*3.33 (*± *1.28)*	*4.17 (*± *0.64)*	*4.20 (*± *0.77)*

**Table 4:** Comparative analysis of the performance of four Large Language Models (OpenBioLLM-8b, BioMistral-7b, ChatGPT-4o, DeepSeek-V3) in answering patient questions before an interventional radiology procedure (BEST, CT-HDR, TAPE)

	**OpenBioLLM-8b**	**BioMistral-7b**	**ChatGPT-4o**	**DeepSeek-V3**	***P*-value Friedman-Test**
**BEST**					
*** All Questions*** ***(Mean, SD)***	3.51 (± 1.15)	2.92 (± 1.35)	4.36 (± 0.71)	4.49 (± 0.77)	< 0.001**
General Information (Mean, SD)	2.88 (± 1.30)	2.50 (± 1.46)	4.71 (± 0.50)	4.12 (± 1.11)	< 0.001**
Preparation/Planning (Mean, SD)	4.10 (± 0.42)	3.30 (± 1.44)	4.50 (± 0.50)	4.60 (± 0.55)	0.186
Risks/Contraindications (Mean, SD)	3.86 (± 1.38)	3.14 (± 1.25)	3.64 (± 1.03)	4.86 (± 0.24)	0.016*
Recovery/Follow-Up (Mean, SD)	4.00 (± 0,58)	2.93 (± 1.40)	4.43 (± 0.53)	4.50 (± 0.58)	0.014*
Side Effects/Complications (Mean, SD)	3.30 (± 1.04)	3.20 (± 1.35)	4.30 (± 0.45)	4.70 (± 0.45)	0.023*
**CT-HDR**					
*** All Questions*** ***(Mean, SD)***	3.32 (± 1.13)	3.03 (± 1.06)	3.81 (± 0.76)	4.24 (± 0.81)	< 0.001**
General Information (Mean, SD)	3.05 (± 1.28)	2.95 (± 0.86)	3.50 (± 0.62)	4.05 (± 0.86)	0.025*
Preparation/Planning (Mean, SD)	3.56 (± 1.13)	3.39 (± 1.11)	3.67 (± 0.61)	4.33 (± 0.97)	0.178
Risks/Contraindications (Mean, SD)	3.10 (± 1.39)	2.40 (± 1.47)	4.40 (± 0,82)	4.40 (± 0.22)	0.007**
Recovery/Follow-Up (Mean, SD)	3.40 (± 1.14)	3.10 (± 0.96)	3.70 (± 1.04)	4.10 (± 0,96)	0.176
Side Effects and Complications (Mean, SD)	3.60 (± 0.74)	3.10 (± 1.08)	4.20 (± 0,67)	4.40 (± 0,82)	0.010*
**TAPE**					
*** All Questions*** ***(Mean, SD)***	3.34 (± 1.16)	3.33 (± 1.28)	4.17 (± 0.64)	4.20 (± 0.77)	< 0.001**
General Information (Mean, SD)	3.00 (± 1.06)	3.83 (± 0.94)	4.44 (± 0.53)	3.94 (± 0.81)	0.037*
Preparation/Planning (Mean, SD)	3.30 (± 1.23)	3.05 (± 1.30)	3.85 (± 0.88)	4.25 (± 0.59)	0.057
Risks/Contraindications (Mean, SD)	3.90 (± 1.08)	2.60 (± 1.71)	4.30 (± 0.57)	3.90 (± 0,96)	0.392
Recovery/Follow-Up (Mean, SD)	3.83 (± 0,75)	3.50 (± 1.05)	4.25 (± 0.42)	4.67 (± 0.61)	0.013*
Side Effects and Complications (Mean, SD)	2.90 (± 1.60)	3.50 (± 1.62)	4.10 (± 0.42)	4.30 (± 0.97)	0.182

*SD *Standard deviation

^*^Significant (*p* < 0.050); **p < 0.010; n.s.: *p* > 0.050

For BEST, DeepSeek-V3 achieved the highest average score [4.49 (± 0.77)], followed by ChatGPT-4o [4.36 (± 0.71)]. OpenBioLLM-8b [3.51 (± 1.15)] and BioMistral-7b [2.92 (± 1.35)] scored significantly lower (*p* < 0.001). Significant differences were observed in General Information (*p* < 0.001), Risks/Contraindications (*p* = 0.016), Recovery/Follow-Up (*p* = 0.014), and Side Effects/Complications (*p* = 0.023), but not in Preparation/Planning (*p* = 0.186).

For CT-HDR brachytherapy, DeepSeek-V3 again outperformed other models [4.24 (± 0.81)], followed by ChatGPT-4o [3.81 (± 0.76)], OpenBioLLM-8b [3.32 (± 1.13)], and BioMistral-7b [3.03 (± 1.06)] (*p* < 0.001). Statistically significant differences were specifically noted in General Information (*p* = 0.025), Risks/Contraindications (*p* = 0.007), and Side Effects/Complications (*p* = 0.010), while Preparation/Planning (*p* = 0.178) and Recovery/Follow-Up (*p* = 0.176) showed no significant differences.

For TAPE, ChatGPT-4o [4.17 (± 0.64)] and DeepSeek-V3 [4.20 (± 0.77)] performed similarly, significantly surpassing OpenBioLLM-8b [3.34 (± 1.16)] and BioMistral-7b [3.33 (± 1.28)] (*p* < 0.001). Significant differences were observed in General Information (*p* = 0.037) and Recovery/Follow-Up (*p* = 0.013). Categories Preparation/Planning (*p* = 0.057), Risks/Contraindications (*p* = 0.392), and Side Effects/Complications (*p* = 0.182) showed no statistically significant differences. Pairwise post-hoc comparisons between the models for all questions are summarized in Table 5.

**Table 5:** Display of *p* values of Wilcoxon signed-rank test with Holm correction for all questions per intervention

***P* values Wilcoxon signed-rank test with Holm correction**				
	*OpenBioLLM-8b*	*BioMistral-7b*	*ChatGPT-4o*	*DeepSeek-V3*
**BEST—**All Questions				
* OpenBioLLM-8b*	**-**	0.073 (n.s.)	0.003 **	< 0.001 **
* BioMistral-7b*	-	-	< 0.001 **	< 0.001 **
* ChatGPT-4o*	-	-	-	0.332 (n.s.)
* DeepSeek-V3*	-	-	-	-
**CT-HDR—**All Questions				
* OpenBioLLM-8b*	**-**	0.133 (n.s.)	0.017 *	0.001 **
* BioMistral-7b*	-	-	0.003 **	< 0.001 **
* ChatGPT-4o*	-	-	-	< 0.010 **
* DeepSeek-V3*	-	-	-	-
**TAPE—**All Questions				
* OpenBioLLM-8b*	**-**	1.000 (n.s.)	0.001 **	0.002 **
* BioMistral-7b*	-	-	0.002 **	0.002 **
* ChatGPT-4o*	-	-	-	1.000 (n.s.)
* DeepSeek-V3*	-	-	-	-

^*^Significant (*p* < 0.050); **p < 0.010; n.s.: *p* > 0.050

**Fig. 2:**
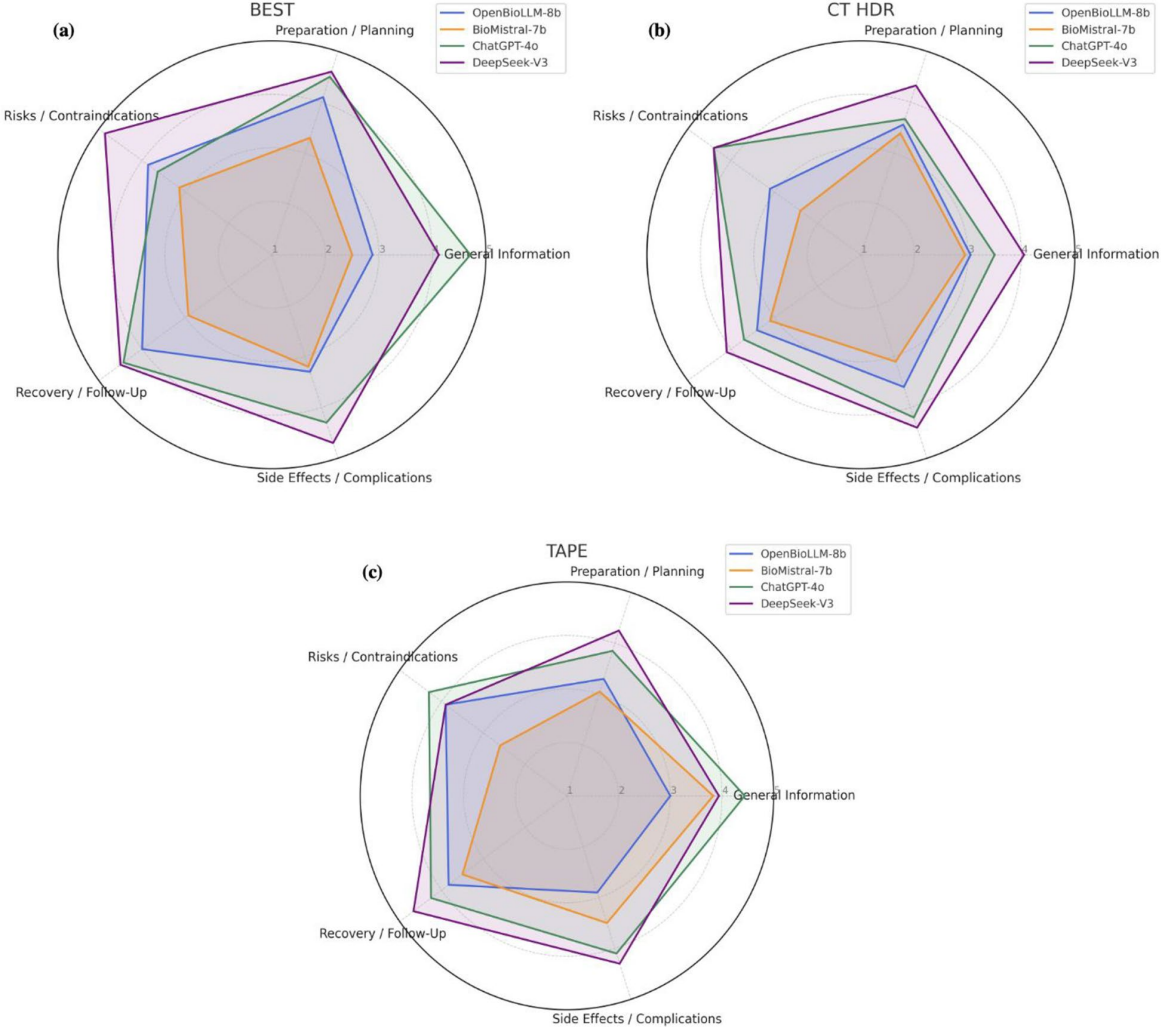
Radar plots that display the illustrate the average performance of four large language models (OpenBioLLM-8b, BioMistral-7b, ChatGPT-4o, DeepSeek-V3) in answering patient questions before interventional radiology procedures **a** BEST, **b** CT HDR, and **c** TAPE across five informational categories

Radar plots in Fig. 2 illustrate average category scores for each procedure. Interrater agreement varied across models and procedures, with the highest consistency for BioMistral-7b (ICC up to 0.69) and the lowest for ChatGPT-4o, particularly for TAPE-related questions with ICC as low as 0.13 (ICC and Cohen’s Kappa in Supplementary Table S2; ratings per radiologist for each procedure are displayed in Supplementary Tables S3–S5).

## Discussion

This study evaluated the performance of four advanced LLMs in addressing potentially relevant and frequently asked patient questions related to TAPE, BEST, and CT-HDR brachytherapy procedures. According to two interventional radiologists, DeepSeek-V3 and ChatGPT-4o demonstrated strong performance by delivering accurate and understandable responses, while the medically pre-trained OpenBioLLM-8b and BioMistral-7b performed significantly worse across all procedures and produced potentially hazardous answers. For instance, OpenBioLLM-8b failed to identify manifest hyperthyroidism as a contraindication, which is concerning given that all three procedures involve iodinated contrast media, which may cause a potentially life-threatening thyrotoxic crisis in patients with hyperthyroidism [29]. BioMistral-7b provided inaccurate information regarding radiation exposure, incorrectly asserting that the radiation dose would be similar to or lower than that of a chest radiograph in all three procedures.

While OpenBioLLM-8b and BioMistral-7b were developed using biomedical training data, it is likely that the limited size and scope of their training datasets, relative to the larger models ChatGPT-4o and DeepSeek-V3, contributed to the inaccurate and potentially harmful responses observed. Responses generated by ChatGPT-4o and DeepSeek-V3 exhibited superior structural organization, enabling step-by-step comprehension of complex medical information.

Beyond the examples described, additional inaccuracies and potentially misleading responses occurred in all categories. While many errors were minor, future analyses would benefit from qualitatively categorizing responses by clinical impact, for example classifying them as benign inaccuracies, misleading but low-risk statements, or potentially harmful content. The evaluated LLMs were trained on broad, general-purpose data, not on procedure-specific or IR-focused material. Accuracy would likely improve with fine-tuning on tailored, domain-specific datasets.

However, the two models showed only low to moderate interrater agreement, underscoring the subjective way LLM responses are interpreted. In contrast, stronger agreement on the lower-performing models could reflect the ease of identifying clearly incorrect or misleading content.

In this study, less commonly performed IR procedures were intentionally selected to evaluate LLM performance under challenging and less-represented clinical settings. Given the evolving nature of IR, assessing how LLMs respond to newer or niche procedures is of significant relevance. Although this focus limits generalizability, it highlights the potential and robustness of LLMs in underrepresented areas.

Several studies have shown that LLMs can provide accurate responses to patient questions in IR [30, 31]. ChatGPT-4 showed strong performance in explaining IR complications but requires health literacy to be fully understood [32]. Combining LLMs with educational videos may enhance patient understanding of IR procedures and satisfaction during informed consent [33]. However, not all studies reported positive conclusions for LLMs in IR patient education, highlighting incomplete or inaccurate patient education content [31]. Hofmann and Vairavamurthy found responses incomplete or lacking depth, especially for risks and alternatives, with more experienced reviewers rating LLMs less favorably [5]. This issue is not exclusive to ChatGPT, as previous studies identified complications in IR cases that had not been documented during the consent process [34]. Several studies report issues with LLMs when answering radiology-related questions, including errors, jargon, and hallucinations. Jeblick et al. [35] found that approximately one-third of simplified radiology reports contained errors potentially harmful to patients. Although patients rated ChatGPT-4.0 superior to medical experts for empathy and usefulness, prior studies indicate that patients frequently struggle to recognize harmful or inaccurate advice [36]. This emphasizes the importance of transparent communication and expert oversight, as specialized medical knowledge remains essential to identify and reduce harmful or misleading information produced by LLMs [37].

The strong performance of DeepSeek-V3 observed in this study aligns with the literature [27, 38, 39], but concerns about user data privacy and regulatory challenges persist [40]. Broader ethical concerns include legal accountability and the protection of human rights [41, 42]. Determining responsibility in cases of patient harm is particularly complex [43], and current data collection methods pose additional threats to patient confidentiality and privacy [44].

Beyond privacy concerns, LLMs could undermine the doctor-patient relationship and reduce trust in medical professionals. However, when appropriately applied, they may improve informed decision-making by providing clear information in complex clinical situations [45]. Despite these benefits, patient safety must remain the priority in future LLM development, reinforcing the urgent need for the establishment of ethical frameworks to guide responsible LLM use [44].

This study compared open-source models (DeepSeek-V3, OpenBioLLM-8b, BioMistral-7b) with the proprietary ChatGPT-4o. Although ChatGPT-4o performed well, concerns remain about transparency and data security risks [46]. Open-source models offer distinct advantages, including enhanced transparency, greater customization opportunities, and increased control over patient data. Given that DeepSeek-V3 is freely accessible and exhibited high performance, it may represent a more practical tool for broader patient access.

Obtaining informed consent in IR is often complex and time-consuming. LLMs could streamline this process, enhance patient empowerment, and improve communication efficiency. However, the complexity of certain IR procedures, including risks associated with off-label device use, demands medical expertise [47]. While DeepSeek-V3 and ChatGPT-4.0 performed well in this study, instances of misinformation produced by the two medically pre-trained LLMs emphasize the continuing need for physician oversight to ensure patient safety [48]. Time savings could be achieved by integrating LLMs into structured clinical pathways with outputs limited to low-risk content, while physician oversight is reserved for high-risk areas or responses flagged as low-confidence by the model. This approach could maintain patient safety without requiring a full review of all outputs. Besides physician oversight, the integration of LLMs into IR workflows could benefit from automated confidence scoring, domain-specific fine-tuning, and incorporation into structured educational platforms like interactive consent tools. Combining LLM outputs with validated multimedia resources may also improve safety and patient comprehension. Further, the implementation should be based on trusted IR guidelines and approved patient information. The data should be protected through secure hosting, access controls, and checks for contraindications.

Future studies should focus on model safety, accuracy, and adherence to clinical guidelines.

## Limitations

The questions were developed by two radiology residents based on clinical experience within a single institution, limiting the representativeness. Although validated by two board-certified interventional radiologists, rewording patient questions may also have altered their original nuance. As we kept a single phrasing per question for consistency, residual sensitivity to alternative phrasings may persist. The assessment covered only three less commonly performed IR procedures, reducing generalizability. LLMs were selected based on public awareness and prior validation, introducing possible selection bias. No patients were involved in the design or evaluation of the questions, and standardized prompting does not fully reflect real doctor-patient interactions. Responses were evaluated by only two board-certified radiologists, introducing potential subjective bias. Future studies should include direct patient evaluation to assess comprehensibility and perceived educational value and thereby better reflect educational impact. As questions or errors were not assigned to risk tiers, future research should categorize responses by clinical relevance and potential harm to better evaluate patient safety. Although the study primarily assessed accuracy and misinformation in LLM-generated responses, aspects like linguistic accessibility and empathetic communication may also matter in patient education. Despite the limitations, the findings provide a valuable foundation for future research, ideally incorporating direct patient participation.

## Conclusions

DeepSeek-V3 and ChatGPT-4o demonstrated a strong performance in answering questions related to TAPE, BEST, and CT-HDR brachytherapy, highlighting their potential for patient education and communication improvement. This is especially relevant in IR, where complex but minimally invasive procedures are often explained within tight consultation windows. OpenBioLLM-8b and BioMistral-7b produced more frequent inaccuracies, underscoring the risks of integrating smaller, biomedical-specific models into clinical practice. These findings demonstrate that LLMs cannot substitute comprehensive medical consultations yet. Nevertheless, LLMs will play an increasing role in radiology and patient care. Future research should validate these findings, incorporate patient feedback, and evaluate LLM integration into clinical workflows, particularly retrieval-augmented and fine-tuned models aligned with IR guidelines.

## Supplementary Information

Supplementary Material 1.

Supplementary Material 2.

## Data Availability

The datasets used and/or analyzed during the current study are available from the corresponding author on reasonable request.

## Abbreviations

LLM: Large language model
CT-HDR: Computed tomography-guided high-dose-rate
BEST: Bleomycin electrosclerotherapy
TAPE: Transarterial periarticular embolization
IR: Interventional radiology
ICC: Intraclass correlation coefficient
