# Supplementary material for: Large language models for patient education prior to interventional radiology procedures: a comparative study
Source: CVIR Endovasc. 2025 Oct 13;8:81. doi: 10.1186/s42155-025-00609-z (PMC12518724; doi:10.1186/s42155-025-00609-z)
Supplement: Supplementary file 1 — Supplementary Material 1. [file 42155_2025_609_MOESM1_ESM.docx]

**Supplementary Material**

**Supplementary Table S1** Likert-scale for radiologist evaluation of LLM response quality (accuracy / correctness / likelihood to mislead)

| **Rating** | **Description** |
| --- | --- |
| 5 | Very accurate / completely correct, very unlikely to mislead |
| 4 | Accurate / mostly correct, only very few inaccuracies, unlikely to mislead |
| 3 | Neutral / moderately accurate, overall acceptable |
| 2 | Inaccurate / mostly false, likely to mislead |
| 1 | Very inaccurate / completely false, very likely to mislead |

**Supplementary Table S2** Display of Interrater Agreement for both radiologists (Intraclass Correlation Coefficient (ICC) and Cohen’s Kappa) per model and interventional procedure

|  | **ICC** | | | **Cohen’s Kappa** | | |
| --- | --- | --- | --- | --- | --- | --- |
| **Model** | *BEST* | *CT HDR* | *TAPE* | *BEST* | *CT HDR* | *TAPE* |
| *BioMistral-7b* | 0.677 | 0.584 | 0.687 | 0.671 | 0.577 | 0.681 |
| *ChatGPT-4o* | 0.371 | 0.189 | 0.126 | 0.364 | 0.185 | 0.123 |
| *DeepSeek-V3* | 0.393 | 0.274 | 0.519 | 0.387 | 0.268 | 0.512 |
| *OpenBioLLM-8b* | 0.466 | 0.61 | 0.693 | 0.459 | 0.603 | 0.687 |

**Supplementary Table S3** Grading of the performance of four Large Language Models Models (OpenBioLLM-8b, BioMistral-7b, ChatGPT-4o, DeepSeek-V3) in answering patient questions before a ***BEST*** procedure

| **Rating** | **OpenBioLLM-8b** | | **BioMistral-7b** | | **ChatGPT-4o** | | **DeepSeek-V3** | |
| --- | --- | --- | --- | --- | --- | --- | --- | --- |
|  | R1 | R2 | R1 | R2 | R1 | R2 | R1 | R2 |
| 5 | 16 (44.44%) | 5 (13.89%) | 12 (33.33%) | 2 (5.56%) | 19 (52.78%) | 20 (55.56%) | 24 (66.67%) | 24 (66.67%) |
| 4 | 10 (27.78%) | 11 (30.56%) | 5 (13.89%) | 9 (25.00%) | 10 (27.78%) | 14 (38.89%) | 8  (22.22%) | 9  (25.00%) |
| 3 | 4 (11.11%) | 9 (25.00%) | 6 (16.67%) | 8 (22.22%) | 5  (13.89%) | 1  (2.78%) | 1  (2.78%) | 2  (5.56%) |
| 2 | 1  (2.78%) | 7 (19.44%) | 3  (8.33%) | 9 (25.00%) | 1  (2.78%) | 1  (2.78%) | 1  (2.78%) | 1  (2.78%) |
| 1 | 5 (13.89%) | 4 (11.11%) | 10 (27.78%) | 8 (22.22%) | 1  (2.78%) | 0  (0.00%) | 2  (5.56%) | 0  (0.00%) |
| Average | 3.86 / 5 | 3.17 / 5 | 3.17 / 5 | 2.67 / 5 | 4.25 / 5 | 4.47 / 5 | 4.42 / 5 | 4.56 / 5 |

*Legend:* R1, radiologist 1; R2, radiologist 2.

**Supplementary Table S4** Grading of the performance of four Large Language Models Models (OpenBioLLM-8b, BioMistral-7b, ChatGPT-4o, DeepSeek-V3) in answering patient questions before a ***CT HDR*** procedure

| **Rating** | **OpenBioLLM-8b** | | **BioMistral-7b** | | **ChatGPT-4o** | | **DeepSeek-V3** | |
| --- | --- | --- | --- | --- | --- | --- | --- | --- |
|  | R1 | R2 | R1 | R2 | R1 | R2 | R1 | R2 |
| 5 | 10 (29.41%) | 0  (0.00%) | 7 (20.59%) | 0 (0.00%) | 6  (17.65%) | 13 (38.24%) | 22 (64.71%) | 13 (38.24%) |
| 4 | 11 (32.35%) | 17 (50.00%) | 5 (14.71%) | 12 (35.29%) | 12 (35.29%) | 14 (41.18%) | 5  (14.71%) | 16 (47.06%) |
| 3 | 3  (8.82%) | 10 (29.41%) | 12 (35.29%) | 13 (38.24%) | 9  (26.47%) | 7 (20.59%) | 3  (8.82%) | 4  (11.76%) |
| 2 | 4 (11.76%) | 4 (11.76%) | 5 (14.71%) | 4 (11.76%) | 5  (14.71%) | 0  (0.00%) | 2  (5.88%) | 1  (2.94%) |
| 1 | 6 (17.65%) | 3 (8.82%) | 5 (14.71%) | 5 (14.71%) | 2  (5.88%) | 0  (0.00%) | 2  (5.88%) | 0  (0.00%) |
| Average | 3.44 / 5 | 3.21 / 5 | 3.12 / 5 | 2.94 / 5 | 3.44 / 5 | 4.18 / 5 | 4.26 / 5 | 4.21 / 5 |

*Legend:* R1, radiologist 1; R2, radiologist 2.

**Supplementary Table S5** Grading of the performance of four Large Language Models Models (OpenBioLLM-8b, BioMistral-7b, ChatGPT-4o, DeepSeek-V3) in answering patient questions before a ***TAPE*** procedure

| **Rating** | **OpenBioLLM-8b** | | **BioMistral-7b** | | **ChatGPT-4o** | | **DeepSeek-V3** | |
| --- | --- | --- | --- | --- | --- | --- | --- | --- |
|  | R1 | R2 | R1 | R2 | R1 | R2 | R1 | R2 |
| 5 | 12 (34.29%) | 3  (8.57%) | 15 (42.86%) | 4 (11.43%) | 11 (31.43%) | 17 (48.57%) | 17 (48.57%) | 15 (42.86%) |
| 4 | 5 (14.29%) | 14 (40.00%) | 5 (14.29%) | 10 (28.57%) | 15 (42.86%) | 15 (42.86%) | 11 (31.43%) | 12 (34.29%) |
| 3 | 8 (22.86%) | 9 (25.71%) | 3  (8.57%) | 13 (37.14%) | 7  (20.00%) | 2  (5.71%) | 5  (14.29%) | 7  (20.00%) |
| 2 | 7 (20.00%) | 6 (17.14%) | 5 (14.29%) | 5 (14.29%) | 1  (2.86%) | 1  (2.86%) | 2  (5.71%) | 1  (2.86%) |
| 1 | 3  (8.57%) | 3  (8.57%) | 7 (20.00%) | 3 (8.57%) | 1  (2.86%) | 0  (0.00%) | 0  (0.00%) | 0  (0.00%) |
| Average | 3.46 / 5 | 3.23 / 5 | 3.46 / 5 | 3.20 / 5 | 3.97 / 5 | 4.37 / 5 | 4.23 / 5 | 4.17 / 5 |

*Legend:* R1, radiologist 1; R2, radiologist 2.

**Supplementary Table S6** Display of p values of Wilcoxon Signed-Rank test with Holm correction for subcategories with significant differences for Friedman test for ***BEST***

| **P values Wilcoxon Signed-Rank test with Holm correction** | | | | |
| --- | --- | --- | --- | --- |
| **BEST** | *OpenBioLLM-8b* | *BioMistral-7b* | *ChatGPT-4o* | *DeepSeek-V3* |
| General Information | | | | |
| *OpenBioLLM-8b* | **-** | 0.574 (n.s.) | **0.024 *** | 0.051 (n.s) |
| *BioMistral-7b* | - | - | **0.020 *** | 0.063 (n.s.) |
| *ChatGPT-4o* | - | - | - | 0.230 (n.s.) |
| *DeepSeek-V3* | - | - | - | - |
| Risks / Contraindications | | | | |
| *OpenBioLLM-8b* | **-** | 0.592 (n.s.) | 0.688 (n.s.) | 0.157 (n.s.) |
| *BioMistral-7b* | - | - | 0.592 (n.s.) | 0.156 (n.s.) |
| *ChatGPT-4o* | - | - | - | 0.156 (n.s.) |
| *DeepSeek-V3* | - | - | - | - |
| Recovery / Follow-Up | | | | |
| *OpenBioLLM-8b* | **-** | 0.262 (n.s.) | 0.262 (n.s.) | 0.262 (n.s.) |
| *BioMistral-7b* | - | - | 0.164 (n.s.) | 0.234 (n.s.) |
| *ChatGPT-4o* | - | - | - | 0.706 (n.s.) |
| *DeepSeek-V3* | - | - | - | - |
| Side Effects / Complications | | | | |
| *OpenBioLLM-8b* | **-** | 0.655 (n.s.) | 0.432 (n.s.) | 0.375 (n.s.) |
| *BioMistral-7b* | - | - | 0.432 (n.s.) | 0.375 (n.s.) |
| *ChatGPT-4o* | - | - | - | 0.410 (n.s.) |
| *DeepSeek-V3* | - | - | - | - |

*: significant (p < 0.050); **: p < 0.010; n.s.: p > 0.050

**Supplementary Table S7** Display of p values of Wilcoxon Signed-Rank test with Holm correction for subcategories with significant differences for Friedman test for ***CT HDR***

| **P values Wilcoxon Signed-Rank test with Holm correction** | | | | |
| --- | --- | --- | --- | --- |
| **CT HDR** | *OpenBioLLM-8b* | *BioMistral-7b* | *ChatGPT-4o* | *DeepSeek-V3* |
| General Information | | | | |
| *OpenBioLLM-8b* | **-** | 0.922 (n.s.) | 0.561 (n.s.) | 0.247 (n.s.) |
| *BioMistral-7b* | - | - | 0.300 (n.s.) | 0.100 (n.s.) |
| *ChatGPT-4o* | - | - | - | 0.300 (n.s.) |
| *DeepSeek-V3* | - | - | - | - |
| Risks / Contraindications | | | | |
| *OpenBioLLM-8b* | **-** | 0.375 (n.s.) | 0.375 (n.s.) | 0.375 (n.s.) |
| *BioMistral-7b* | - | - | 0.375 (n.s.) | 0.375 (n.s.) |
| *ChatGPT-4o* | - | - | - | 1.000 (n.s.) |
| *DeepSeek-V3* | - | - | - | - |
| Side Effects / Complications | | | | |
| *OpenBioLLM-8b* | **-** | 0.395 (n.s.) | 0.353 (n.s.) | 0.353 (n.s.) |
| *BioMistral-7b* | - | - | 0.353 (n.s.) | 0.353 (n.s.) |
| *ChatGPT-4o* | - | - | - | 0.414 (n.s.) |
| *DeepSeek-V3* | - | - | - | - |

*: significant (p < 0.050); **: p < 0.010; n.s.: p > 0.050

**Supplementary Table S8** Display of p values of Wilcoxon Signed-Rank test with Holm correction for subcategories with significant differences for Friedman test for ***TAPE***

| **P values Wilcoxon Signed-Rank test with Holm correction** | | | | |
| --- | --- | --- | --- | --- |
| **CT HDR** | *OpenBioLLM-8b* | *BioMistral-7b* | *ChatGPT-4o* | *DeepSeek-V3* |
| General Information | | | | |
| *OpenBioLLM-8b* | **-** | 0.264 (n.s.) | 0.105 (n.s.) | 0.239 (n.s.) |
| *BioMistral-7b* | - | - | 0.301 (n.s.) | 0.777 (n.s.) |
| *ChatGPT-4o* | - | - | - | 0.239 (n.s.) |
| *DeepSeek-V3* | - | - | - | - |
| Recovery / Follow-Up | | | | |
| *OpenBioLLM-8b* | **-** | 0.395 (n.s.) | 0.395 (n.s.) | 0.188 (n.s.) |
| *BioMistral-7b* | - | - | 0.375 (n.s.) | 0.188 (n.s.) |
| *ChatGPT-4o* | - | - | - | 0.387 (n.s.) |
| *DeepSeek-V3* | - | - | - | - |

*: significant (p < 0.050); **: p < 0.010; n.s.: p > 0.050
